# Stakeholder Priorities for Strengthening Sports Medicine Services in Kazakhstan: A Cross-Sectional Health-System Survey

**DOI:** 10.3390/healthcare14193264

**Published:** 2026-10-02

**Authors:** Sabina Muzrapova, Nurlan Jainakbayev, Rashid Sapargaliyev, Murat Umurzakov, Saule Esengarayeva, Nurlan Baisynov

**Affiliations:** 1 Department of Public Health, NEI “ Kazakhstan-Russian Medical University”, Almaty 050000, Kazakhstan; sabina.muzrapova@mail.ru (S.M.); rector@medkrmu.kz (N.J.); rashidsapargaliyev@gmail.com (R.S.); 2 Physical Medicine and Rehabilitation Unit, NEI “ Kazakhstan-Russian Medical University”, Almaty 050000, Kazakhstan; saulessen@gmail.com; 3 Department of Scientific Research, NEI “ Kazakhstan-Russian Medical University”, Almaty 050000, Kazakhstan; nurlan050203@mail.ru

**Keywords:** sports medicine, rehabilitation, injury prevention, priority setting, stakeholder survey, healthcare management, athlete health, sports healthcare systems, Kazakhstan

## Abstract

**Background/Objectives:** Sports medicine systems require evidence-informed prioritization of medical, rehabilitation, preventive, and organizational interventions. This study aimed to assess and rank stakeholders’ perceived importance of proposed development domains for sports medicine in Kazakhstan, rather than the current performance or quality of the existing system. **Methods:** An anonymous cross-sectional survey was conducted between December 2025 and January 2026 among 100 stakeholders involved in medical care, rehabilitation, diagnostics, coaching and training, athletic participation, or sports administration. Twelve development domains were rated on a five-point priority scale, followed by a Top-5 selection task. Because the survey form did not technically enforce selection of exactly five options, the primary Top-5 analysis was restricted to the 47 respondents who completed the task as instructed. **Results:** Among these 47 respondents, rehabilitation and recovery programs were selected by 30/47 (63.8%), injury prevention by 26/47 (55.3%), and both accessibility of medical support and financing/sustainability by 24/47 (51.1%). The all-questionnaire sensitivity ranking (*N* = 100) showed strong but incomplete agreement with the primary exact-five ranking (Spearman’s *ρ* = 0.889; Kendall’s *τ* = 0.762). The 12-domain ratings showed a pronounced ceiling/invariant-response pattern, and subgroup analyses were treated as exploratory because of small and compositionally unequal subgroups. **Conclusions:** The findings describe perceived priorities within the surveyed non-probability stakeholder sample. Given the purposive, snowball-assisted recruitment strategy and the lack of full psychometric validation of the study-specific questionnaire, they should not be interpreted as nationally representative estimates of service quality, current shortages, or national policy priorities.

## 1. Introduction

Sports medicine supports athlete health through prevention, clinical assessment, rehabilitation, safe return to sport, and monitoring of training and recovery. The international literature addresses these functions through integrated athlete-health models, training-load monitoring, injury surveillance, and health-system frameworks [1,2,3,4,5]. However, the relative importance assigned to individual development domains is context-dependent, and priorities identified in one health or sport system cannot be assumed to apply directly to another.

In Kazakhstan, sports medicine is embedded in both healthcare and sports-sector regulatory frameworks. Article 43 of the Law of the Republic of Kazakhstan ‘On Physical Culture and Sports’ defines medical support as including systematic health monitoring, assessment of the adequacy of physical loads, prevention and treatment of disease and injury, medical rehabilitation, decisions on admission to training and competition, first aid, and other athlete-health functions [6]. Amendments introduced in 2025 also established a National Organization for Sports Medicine and Rehabilitation as a state-owned enterprise with responsibilities that include specialized athlete care, medical examinations and comprehensive functional testing, rehabilitation and recovery, professional training, and medical support for national teams and sporting events [6].

The operational framework is further specified by national rules governing medical support during competitions and training activities, as well as recovery after intense physical exertion, illness, and injury [7]. Separate rules regulate athlete medical examinations and distinguish in-depth, staged, current, comprehensive functional, and pre-competition assessments [8]. These regulations indicate that sports medicine in Kazakhstan has a formal organizational and clinical framework extending from medical clearance and event coverage to rehabilitation and functional monitoring.

Financing is also linked to the public sports system. Current rules for high-performance sport provide for financing from republican and local budgets and explicitly include medical support and medical care among eligible expenditure categories [9]. This creates a formal mechanism for resourcing medical support in high-performance sport, although publicly available evidence remains limited regarding the adequacy, distribution, and consistency of such resources across sport levels and regions.

At the same time, national policy documents have identified structural constraints. The Concept for the Development of Physical Culture and Sports of the Republic of Kazakhstan for 2023–2029 reports shortages of sports-medicine personnel and gaps in infrastructure, equipment, and methodological support. Its workforce estimates indicated 24 sports physicians available against an estimated requirement of 48 for national teams; no sports psychologists, dietitians, or nutritionists were listed as available against estimated requirements of 15, nine, and eight, respectively [10]. Earlier official government reporting also documented uneven regional availability of dedicated sports-medicine organizations, including six regions without such organizations at that time [11]. These sources describe system-level challenges independently of the present survey and provide a Kazakhstan-specific rationale for examining stakeholder priorities.

Despite this regulatory framework and documented development needs, empirical evidence on how different Kazakhstan stakeholders prioritize specific domains of sports medicine development remains limited. International studies provide important evidence on integrated athlete-health management, rehabilitation, injury prevention, return-to-sport decision-making, training-load monitoring, and multidisciplinary athlete support [1,2,3,12,13,14,15,16,17], but they cannot establish which organizational or clinical domains stakeholders in Kazakhstan consider most important for future development.

Accordingly, the present study was designed as a stakeholder priority-setting survey. The 12 domains represent distinct proposed areas for development, including accessibility, safety clearance, standardized examination protocols, return-to-play, rehabilitation, injury prevention, workload monitoring, staffing, infrastructure, digitalization, intersectoral collaboration, and financing. The ratings are interpreted as perceived importance or priority and not as measures of the current performance, availability, quality, or deficiency of services. The study does not attempt to validate a national tiered service model; any such framework would require separate level-specific evaluation using representative samples.

The primary objective was to assess and rank stakeholder-perceived priorities for sports medicine development in mass sport, youth reserve sport, and elite sport in Kazakhstan. Secondary analyses explored whether domain ratings or Top-5 selections differed according to professional group, level of sport involvement, or professional experience. These subgroup analyses were intended to identify potential heterogeneity in stakeholder perspectives rather than to produce nationally representative estimates or definitive policy recommendations.

## 2. Materials and Methods

### 2.1. Study Design and Primary Estimand

This cross-sectional analytical stakeholder survey evaluated the perceived importance of proposed development domains for sports medicine in Kazakhstan. The primary estimand was the priority assigned by respondents to each proposed development domain, not the current performance, quality, availability, or deficiency of the existing sports medicine system. The unit of observation was the individual respondent.

### 2.2. Participants, Recruitment, and Representativeness

The analytical sample comprised 100 stakeholders involved in medical care, rehabilitation, diagnostics, coaching and training, athletic participation, or sports administration. Eligible participants included physicians and medical specialists, physiotherapy and rehabilitation specialists, coaches, athletes, and managers or administrators with relevant professional or practical involvement.

Participants are referred to throughout the revised manuscript as stakeholders rather than experts because no uniform credential- or experience-based definition of expert status was applied. Recruitment used purposive sampling with an additional snowball component. The questionnaire was initially distributed directly to 200 potentially eligible individuals, and recipients could forward the survey link within their professional networks. Because an individual-level recruitment source was not recorded, the number of analyzed respondents recruited by direct invitation versus snowball forwarding could not be reconstructed, and the total number of individuals exposed to forwarded links was unknown. Accordingly, a conventional overall response rate could not be calculated, and the sample was not intended to provide nationally representative estimates.

Respondents were recruited from across Kazakhstan, but regional affiliation was not collected as an analytical variable. Consequently, regional representativeness and regional differences could not be assessed.

The inclusion criteria were professional, clinical, organizational, athletic, or practical involvement in sports medicine, rehabilitation, athlete support, coaching, functional diagnostics, professional sport, or sports management; experience or participation in mass sport, youth reserve sport, or elite sport; and voluntary informed consent. Refusal to participate was an exclusion criterion.

### 2.3. Questionnaire Development and Administration

Data were collected using a study-specific structured questionnaire. The questionnaire contained socio-professional items, 12 proposed development domains rated on a five-point priority scale, a Top-5 selection task, and two open-ended questions. For all 12 Likert-type items, respondents were explicitly instructed to rate priority, where 1 indicated the lowest priority, and 5 indicated the highest priority. The items therefore measured perceived importance or priority rather than respondents’ assessment of current system performance. The complete questionnaire wording, response anchors, Top-5 options, and original Russian-language version are reproduced in Supplementary Table S1.

The 12 domains were developed by the study team to represent major organizational, preventive, rehabilitation, diagnostic, workforce, infrastructure, digital, coordination, and financing components of sports medicine. The questionnaire underwent pilot review for clarity, wording, logical sequence, and transitions between blocks, after which ambiguous wording was refined. The archived study documentation did not contain a recoverable record of the number or characteristics of pilot participants or a formal content-validity index. Accordingly, the questionnaire is treated as a study-specific survey instrument, and no claim of formal content validation is made. The instrument has not undergone full psychometric validation; factorial structure, construct validity, test-retest reliability, and discriminatory performance were not established in this study.

The survey was administered in Russian using Google Forms (Google LLC, Mountain View, CA, USA; web-based version, accessed December 2025–January 2026) between December 2025 and January 2026. The Top-5 item was marked as required; however, the form configuration did not technically enforce selection of exactly five options. As a result, respondents could submit fewer or more than five selections. The archived study documentation does not allow verification of whether the Google Forms “Limit to 1 response” setting or mandatory Google-account sign-in was enabled; therefore, the use of technical measures to prevent repeat participation cannot be confirmed.

### 2.4. Data Completeness, Duplicate Checks, and Top-5 Compliance

All 100 submitted questionnaires included valid responses to each of the 12 priority-rating items; no missing or invalid values were detected across these items. Both open-ended questions were answered by all 100 respondents. No partially completed questionnaires were submitted, and none were excluded for incompleteness before the creation of the final *N* = 100 analytical dataset. No duplicate timestamps or exact duplicate response profiles were identified in the validated analytical dataset. Because no personal identifiers were collected and the platform-level duplicate-prevention settings could not be retrospectively verified, identity-based duplicate verification was not possible; duplicate assessment was therefore limited to the available timestamps and response patterns.

Exactly five Top-5 options were selected by 47 respondents, whereas 53 selected a different number of options. Because the deviation affected the Top-5 task rather than the 12 individual priority ratings, these 53 respondents were retained for respondent characteristics and domain-specific Likert analyses but excluded from the primary Top-5 ranking. Their Top-5 selections were retained only for sensitivity analysis. The complete distribution of the number of selected Top-5 options is provided in the Supplementary Materials.

### 2.5. Professional-Role Recoding

Nineteen respondents reported more than one original professional role. For analytical grouping, roles were mapped to five predefined categories: physician/medical specialist, physiotherapy/rehabilitation, coach, athlete, and manager/administrator. If all selected roles mapped to the same analytical category, the respondent remained in that category. If the reported roles spanned more than one analytical category, the respondent was classified as having multiple professional groups. Twelve respondents met this latter definition and were excluded only from profession-based inferential comparisons. One psychologist/other respondent was also excluded from profession-based inferential testing because a group of *n* = 1 is not suitable for such comparisons. The resulting profession-based inferential sample contained 87 respondents: 31 physicians, 25 physiotherapy/rehabilitation specialists, 14 athletes, 10 coaches, and 7 managers/administrators.

### 2.6. Outcomes and Handling of the 12 Domains

The 12 domain ratings were analyzed as separate ordinal outcomes. Although internal consistency was explored, the domains are conceptually distinct, and no evidence of unidimensionality or factorial structure was available. Consequently, a total score and mean composite score were not used as primary or secondary inferential outcomes in the revised analysis. Cronbach’s alpha was retained only as supplementary descriptive information on response consistency and was not used to justify a single composite scale. Given the pronounced ceiling and invariant all-maximum response patterns, internal-consistency coefficients were interpreted cautiously because response concentration may increase inter-item correlations and reduce discrimination among domains.

For the Top-5 task, the primary analysis was restricted to the 47 respondents who followed the instruction and selected exactly five domains. The analysis of all 100 questionnaires was treated as a sensitivity analysis only. For Top-5 proportions, 95% Wilson confidence intervals were calculated. Agreement between the primary and sensitivity rankings across the 12 domains was quantified using Spearman’s *ρ* and Kendall’s *τ*, with ties retained in the rankings.

### 2.7. Sample Size and Precision

No formal a priori power calculation aligned with the final domain-specific and subgroup analyses was performed before data collection; therefore, the previous item-to-respondent rule was removed and no retrospective a priori calculation was claimed. For descriptive context only, under a simple binomial approximation at *p* = 0.50, the nominal 95% confidence-interval half-width was approximately ±9.8 percentage points for the full sample (*N* = 100) and ±14.3 percentage points for the primary exact-five Top-5 sample (*n* = 47). Because recruitment was non-probability based, these figures describe nominal achieved precision only; they are not design-based margins of error and do not constitute prospective sample-size justification.

Given several small subgroups, subgroup analyses were treated as exploratory in the revised analysis and interpreted with an emphasis on effect sizes, multiplicity adjustment, and uncertainty.

### 2.8. Statistical Analysis

Statistical analyses were conducted in R version 4.5.2 (R Foundation for Statistical Computing, Vienna, Austria). The revised analyses used the R packages readxl, dplyr, tidyr, purrr, stringr, tibble, rstatix, psych, openxlsx, and ggplot2.

Categorical variables are reported as *n* (%) without routine percentage standard errors. Domain ratings are summarized using the median and interquartile range [Q1–Q3] because they are ordinal five-point responses.

For comparisons of each of the 12 domain ratings across three or more independent groups, the Kruskal–Wallis test was used. Epsilon-squared (*ε*^2^) was reported as the omnibus effect size. For omnibus tests with unadjusted *p* < 0.05, 95% bootstrap confidence intervals for *ε*^2^ were estimated from 2000 stratified bootstrap resamples. Within each grouping factor, the Benjamini–Hochberg adjustment was applied across the 12 domain-level omnibus tests. Dunn pairwise comparisons were generated after an omnibus Kruskal–Wallis test with unadjusted *p* < 0.05 and were adjusted using the Holm procedure. Complete omnibus and pairwise results, including adjusted *p*-values, are provided in the Supplementary Materials.

Top-5 subgroup comparisons were performed only in the primary exact-five sample (*n* = 47). Pearson’s chi-square test was used when expected cell counts were adequate; otherwise, Fisher’s exact test with Monte Carlo *p*-values based on 100,000 replicates was used. Cramer’s *V* was reported as the association effect size, and Benjamini–Hochberg adjustment was applied across the 12 Top-5 domain tests within each grouping factor. Because the primary exact-five sample was small, professional-role comparisons were restricted to groups with at least five respondents.

To assess whether the marked experience-group differences could reflect subgroup composition, associations of professional experience with level of sport and with analytical professional group were examined using Fisher’s exact test with Monte Carlo *p*-values based on 100,000 replicates and Cramer’s *V*. All tests were two-sided, with *p* < 0.05 used as the nominal significance threshold; adjusted *p*-values were used for multiplicity-controlled interpretation.

### 2.9. Open-Ended Questions

The questionnaire contained two open-ended questions on perceived problems and priority measures, and all 100 respondents answered both questions. Because the original coder-level audit trail and denominator definition could not be reproduced with sufficient certainty, thematic percentages, qualitative frequency claims, and representative quotations were not retained in the revised Results, Discussion, or Conclusions. A separate qualitative re-analysis using an explicitly documented coding protocol and independent coders would be required before such findings are reinstated.

Because the study had a cross-sectional design, statistical relationships were interpreted as associations rather than causal relationships.

### 2.10. Ethical Considerations

The study was conducted in accordance with the Declaration of Helsinki and the principles of voluntary participation, anonymity, and confidentiality. The project was reviewed and approved by the Local Ethics Committee of the non-profit educational organization ‘Kazakhstan-Russian Medical University’ (protocol No. 22, 22 April 2024). Before participation, respondents were informed about the purpose of the study, the anonymous nature of data collection, and the intended scientific use of the data. Completion and submission of the questionnaire were considered confirmation of voluntary informed consent.

## 3. Results

### 3.1. Respondent Characteristics and Data Quality

All 100 questionnaires in the validated analytical dataset were included in the domain-specific analyses. After recoding professional roles for analysis, 31 respondents were classified as physicians, 25 as physiotherapy/rehabilitation specialists, 14 as athletes, 10 as coaches, seven as managers/administrators, 12 as spanning multiple professional groups, and one as psychologist/other. The sample included 33 stakeholders involved in mass sport, 27 in youth reserve sport, and 40 in elite sport. Professional experience was <1 year for four respondents, 1–3 years for 28, 4–7 years for 28, 8–15 years for 22, and >15 years for 18.

Nineteen respondents reported multiple original professional roles; 12 of these spanned more than one analytical professional group. No missing or invalid values were present across the 12 priority items, and no duplicate timestamps or exact duplicate response profiles were detected in the validated dataset. Twenty-five respondents rated all 12 domains as 5. Exactly five Top-5 choices were submitted by 47 respondents, whereas 53 did not follow the exact-five instruction (Table 1).

### 3.2. Domain-Specific Priority Ratings, Ceiling Effects, and Invariant Response Patterns

Median priority ratings were high across the 12 domains.

Six domains had a median of 5.00 with Q1 = 4.00, while digitalization/database management, intersectoral collaboration, and financing/sustainability also had a median of 5.00 but a lower first quartile of 3.00. Accessibility of medical support and monitoring of training load/recovery had medians of 4.00 [4.00–5.00], and return-to-play protocols had a median of 4.50 [3.75–5.00].

Ceiling and invariant-response effects were substantial: 53.25% of all item-level ratings were equal to 5, and 25 of 100 respondents assigned a score of 5 to all 12 domains. The latter all-maximum pattern is compatible with straight-lining, although the available data cannot distinguish a response-style effect from genuinely uniform judgments that all domains were of maximum priority. Regardless of cause, the upper-bound concentration and within-respondent invariance reduce the discriminatory capacity of the five-point ratings, may increase inter-item correlations, and may attenuate subgroup contrasts. These limitations were therefore considered when interpreting internal consistency and exploratory subgroup analyses (Table 2).

### 3.3. Primary Top-5 Prioritization (Exact-Five Sample, n = 47) and All-Questionnaire Sensitivity Analysis

**Figure 1:**
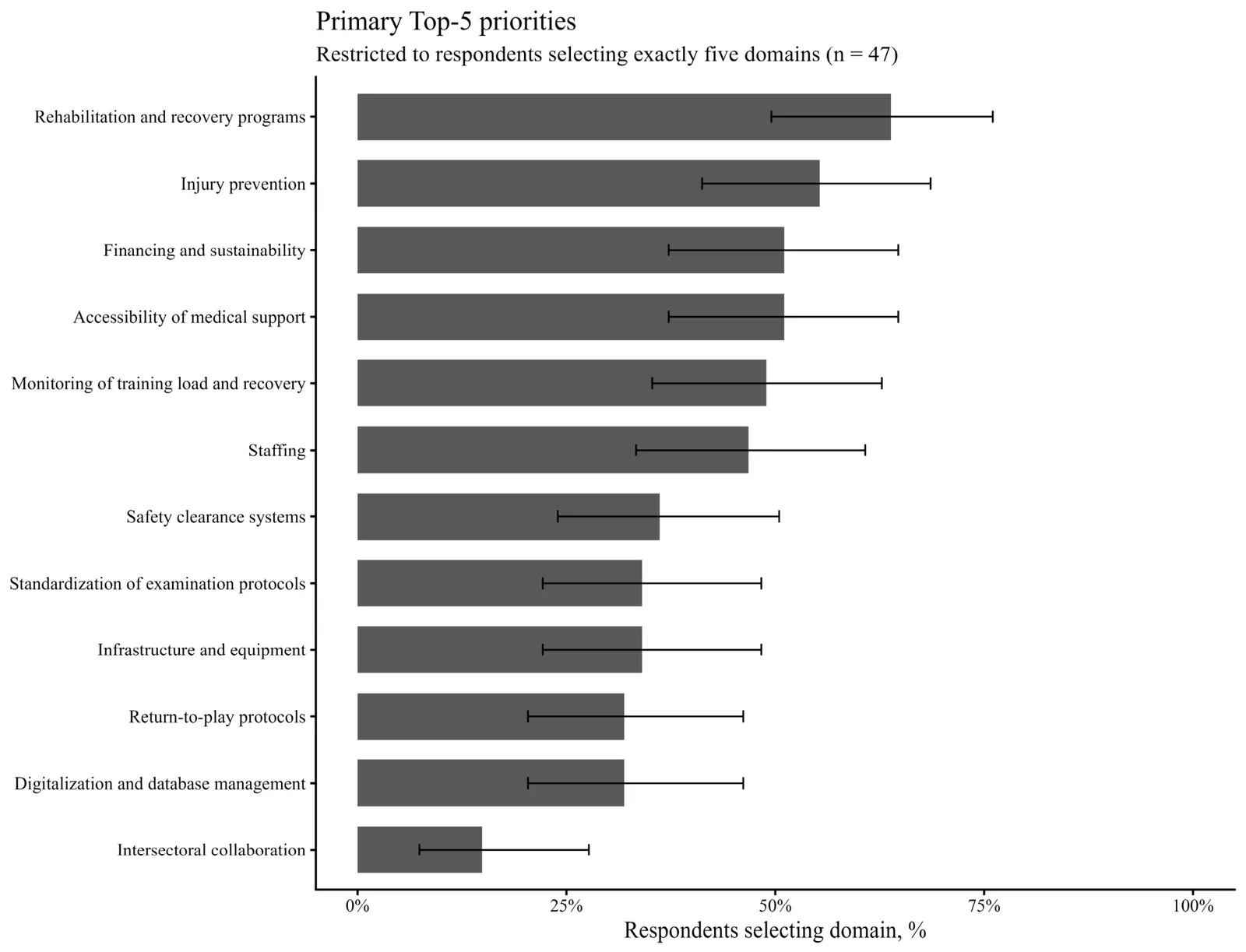
Primary Top-5 priorities among respondents who selected exactly five domains (*n* = 47). Bars show observed proportions and error bars show 95% Wilson confidence intervals.

The primary Top-5 analysis was restricted to the 47 respondents who selected exactly five domains. Rehabilitation and recovery programs ranked first, selected by 30/47 respondents (63.8%, 95% Wilson CI 49.5–76.0%). Injury prevention ranked second (26/47, 55.3%, 95% CI 41.2–68.6%). Accessibility of medical support and financing/sustainability were tied for third (24/47 each, 51.1%, 95% CI 37.2–64.7%), followed by monitoring of training load/recovery (23/47, 48.9%) and staffing (22/47, 46.8%) (Figure 1 and Table 3).

The all-questionnaire Top-5 sensitivity analysis (*N* = 100) showed strong but incomplete agreement with the primary exact-five ranking (*n* = 47). Spearman’s *ρ* was 0.889 (*p* < 0.001), and Kendall’s *τ* was 0.762 (*p* < 0.001). Rehabilitation remained rank 1, and accessibility remained rank 3, but several middle-ranked domains shifted materially. Financing moved from rank 3 in the primary exact-five sample to rank 5 in the all-questionnaire sensitivity analysis, monitoring of training load/recovery moved from rank 5 to rank 8, and safety clearance moved from rank 7 to rank 4. Therefore, the sensitivity analysis supports partial consistency of the leading profile rather than complete stability of the overall ranking. Full sensitivity results and rank shifts are shown in Supplementary Tables S5 and S6.

### 3.4. Exploratory Internal-Consistency Assessment

Cronbach’s alpha for the 12 domain ratings was 0.944, the standardized alpha was 0.945, and the average inter-item correlation was 0.590. These values indicate high internal consistency of the response patterns but do not establish unidimensionality or full psychometric validity. In view of the marked ceiling and invariant all-maximum response patterns, the coefficients may partly reflect restricted response variability or common response style and should not be interpreted as evidence that the instrument can finely discriminate among highly prioritized domains. In accordance with the revised analytical strategy, no total or mean composite score was used for inference. Exploratory internal-consistency information is reported in Supplementary Table S12.

### 3.5. Exploratory Subgroup Analyses of Domain Ratings

Profession-based comparisons included 87 respondents after the exclusion of 12 multiple-group respondents and the psychologist/other respondents. None of the 12 domain ratings differed significantly across the five professional groups after Benjamini–Hochberg correction. Similarly, no domain-level difference across mass sport (*n* = 33), youth reserve sport (*n* = 27), and elite sport (*n* = 40) remained statistically significant after multiplicity correction. Infrastructure/equipment had an unadjusted Kruskal–Wallis *p* = 0.021, but the adjusted *p*-value was 0.254 and was therefore not interpreted as significant.

Professional experience was associated with differences across a broader range of domain-specific priority ratings. After Benjamini–Hochberg correction, 11 of the 12 domains differed across the five experience groups; monitoring of training load/recovery was the only domain that did not remain significant (*H* = 7.270, adjusted *p* = 0.122, *ε*^2^ = 0.034). The largest effect sizes were observed for financing/sustainability (*ε*^2^ = 0.186, bootstrap 95% CI 0.069–0.382), accessibility of medical support (*ε*^2^ = 0.150, 95% CI 0.051–0.331), staffing (*ε*^2^ = 0.148, 95% CI 0.039–0.347), injury prevention (*ε*^2^ = 0.125, 95% CI 0.017–0.338), infrastructure/equipment (*ε*^2^ = 0.121, 95% CI 0.025–0.311), and digitalization/database management (*ε*^2^ = 0.113, 95% CI 0.024–0.308) (Table 4).

Dunn–Holm post hoc comparisons indicated that the significant pairwise contrasts predominantly involved comparisons with the >15-year group. For example, the >15-year group had lower median ratings than the 8–15-year group for accessibility of medical support (3.00 [3.00–4.75] vs. 5.00 [4.00–5.00], Holm-adjusted *p* = 0.008), return-to-play protocols (3.50 [3.00–4.00] vs. 5.00 [4.25–5.00], Holm-adjusted *p* = 0.007), and several other domains. For financing/sustainability, the >15-year median was 2.00 [2.00–3.00] compared with 5.00 [4.00–5.00] in the 1–3-year group and 5.00 [4.25–5.00] in the 8–15-year group; both Holm-adjusted *p*-values were <0.001. Complete pairwise comparisons, including all adjusted *p*-values, are reported in Supplementary Table S8.

**Figure 2:**
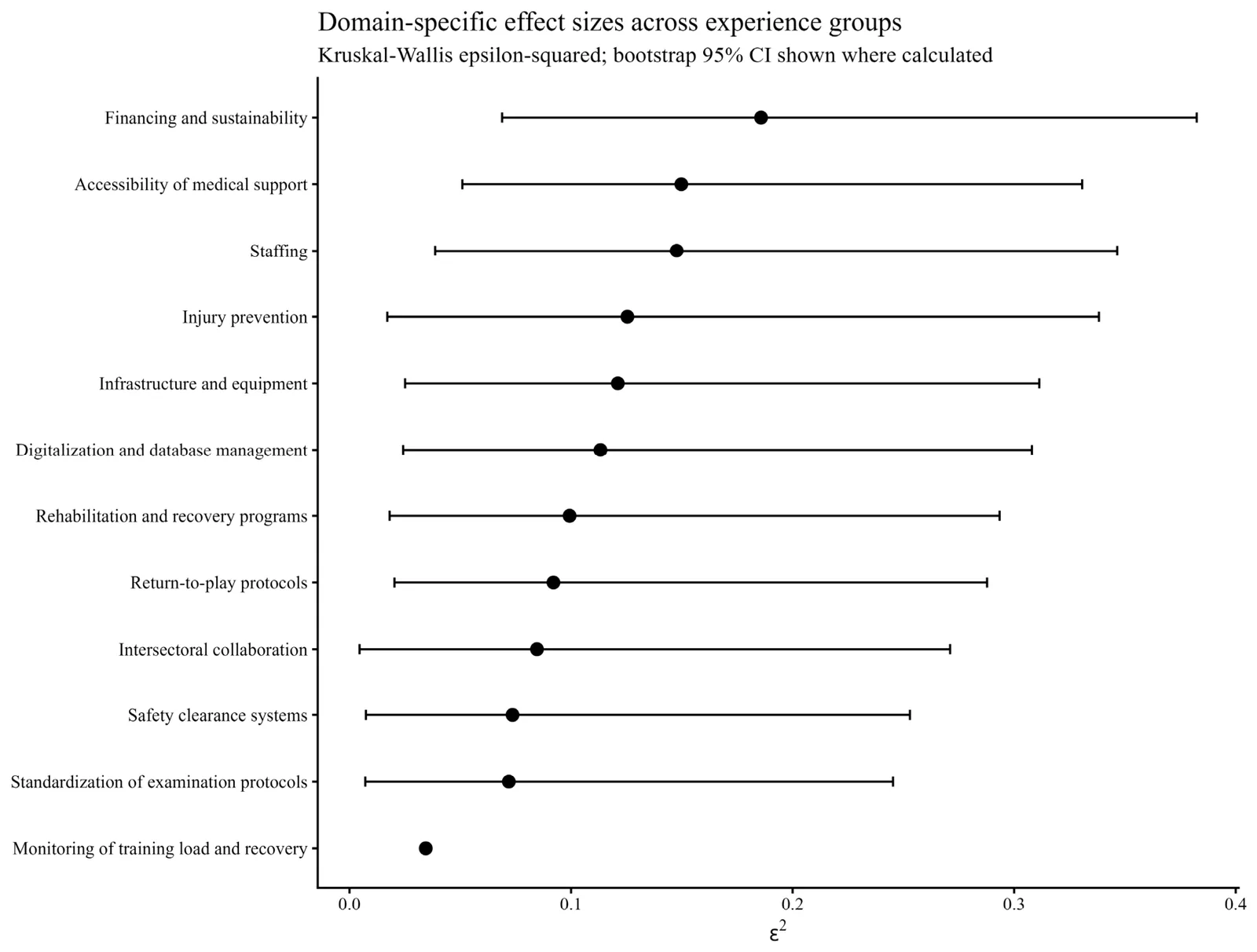
Domain-specific Kruskal–Wallis epsilon-squared effect sizes across professional-experience groups. Bootstrap 95% confidence intervals are shown for omnibus tests with unadjusted *p* < 0.05.

The experience-group findings should not be interpreted as evidence that highly experienced respondents were more critical or more aware of system deficiencies. Experience was associated with level of sport (Fisher’s exact test with Monte Carlo simulation, *p* = 0.0022; Cramer’s *V* = 0.331), and 12 of the 18 respondents with >15 years of experience (66.7%) were involved in elite sport. The association between experience and analytical professional group was not statistically significant (*p* = 0.102; Cramer’s *V* = 0.250). These results indicate potential compositional confounding and support cautious, exploratory interpretation (Figure 2).

### 3.6. Exploratory Top-5 Subgroup Analyses

Exploratory Top-5 subgroup testing was conducted only in the primary exact-five sample (*n* = 47). For profession, four groups met the minimum size criterion: physicians (*n* = 14), physiotherapy/rehabilitation specialists (*n* = 14), athletes (*n* = 8), and coaches (*n* = 5), giving *n* = 41 for this comparison. The primary exact-five sample contained 11 mass-sport, 14 youth-reserve, and 22 elite-sport respondents. For experience, the exploratory categories were collapsed to ≤3 years (*n* = 18), 4–7 years (*n* = 11), 8–15 years (*n* = 9), and >15 years (*n* = 9). No Top-5 domain showed a statistically significant subgroup association after Benjamini–Hochberg correction for professional group, sport level, or experience. Given the small subgroup counts and limited precision, these null findings should not be interpreted as evidence of subgroup equivalence or homogeneity. Complete tests and Cramer’s *V* estimates are provided in Supplementary Table S11.

### 3.7. Open-Ended Response Completion

All 100 respondents answered each of the two open-ended questions. Because the original coder-level audit trail and denominator definition could not be reproduced from the archived materials, thematic frequencies and qualitative quotations were not used in the revised Results.

## 4. Discussion

The revised interpretation of this survey is limited to stakeholder-perceived priorities and does not treat the ratings as evidence of the current performance or quality of Kazakhstan’s sports medicine system. Among the 47 respondents included in the primary exact-five Top-5 analysis, rehabilitation/recovery was most frequently selected, followed by injury prevention, while accessibility of medical support and financing/sustainability were tied for the next-highest selection frequency. These findings indicate what the surveyed stakeholders considered important for future development; they do not independently demonstrate that the corresponding services are currently deficient.

The prominence of rehabilitation and recovery is consistent with the international sports-medicine literature emphasizing integrated athlete-health management, rehabilitation-linked return-to-sport decision-making, and longitudinal health protection [1,15,18]. Rehabilitation in sport is increasingly understood as a staged process that links clinical recovery with functional readiness and the safe return to training and competition. The present findings are therefore consistent with this broader international pattern, while the magnitude of the priority assigned to it remains specific to this stakeholder sample.

Injury prevention was also frequently prioritized. Evidence from systematic reviews and implementation research supports structured preventive programs, neuromuscular training, load management, and context-sensitive implementation in different sport settings [2,16,19,20,21,22]. These external findings support the relevance of injury prevention as a development domain, but they should not be used to infer the current adequacy or inadequacy of preventive services in Kazakhstan from the present ratings alone.

The Kazakhstan-specific policy context provides an independent basis for interpreting why workforce, infrastructure, access, and financing may be salient development topics. National policy documents have reported shortages of sports-medicine personnel and gaps in infrastructure, equipment, and methodological support [10], while earlier official reporting documented uneven regional availability of dedicated sports-medicine organizations [11]. Current legislation has also strengthened the organizational role of the National Organization for Sports Medicine and Rehabilitation and formalized public financing mechanisms for medical support in high-performance sport [6,9]. Importantly, these external sources—not the survey ratings themselves—constitute evidence about system organization and documented constraints.

The Top-5 data also require caution because 53% of respondents did not select exactly five options. Because more than half of the respondents did not complete the task as instructed, this procedural problem substantially constrains the robustness, precision, and interpretability of the resulting priority ranking, even though the sensitivity analysis provides a useful check on ranking consistency. The primary exact-five sample (*n* = 47) therefore provides a methodologically cleaner representation of the intended task. Comparison with the all-questionnaire sensitivity ranking (*N* = 100) showed strong but incomplete agreement: rehabilitation remained the leading domain, but financing, monitoring of training load/recovery, and safety clearance changed by several rank positions. The appropriate interpretation is therefore partial consistency of the leading profile rather than complete stability of the priority hierarchy.

The five-point domain ratings showed a pronounced upper-end concentration: 53.25% of all item-level responses were 5, and 25 of 100 respondents selected the maximum score for all 12 domains. This invariant all-maximum pattern is compatible with straight-lining or another response-style effect, although the available data cannot distinguish such behavior from genuinely uniform judgments that all domains were of very high priority. Regardless of its cause, the combination of ceiling effects and within-respondent invariance reduces the instrument’s discriminatory capacity between domains, may increase inter-item correlations and Cronbach’s alpha, and may reduce sensitivity to subgroup differences. The internal-consistency coefficients should therefore be regarded as descriptive only, and the domain-specific ratings and primary exact-five Top-5 task are more interpretable than an aggregate score. The study-specific questionnaire has not undergone full psychometric validation; factorial structure, construct validity, test-retest reliability, and discriminatory performance remain to be established.

Differences according to professional experience should likewise be interpreted as exploratory. Respondents with more than 15 years of experience provided lower priority ratings across multiple domains, but the cross-sectional data cannot establish why. In the revised analysis, professional experience was associated with the level of sport involvement, and two-thirds of the >15-year group were involved in elite sport. This compositional imbalance, together with the very small <1-year group, makes confounding and imprecision plausible. The earlier interpretation that highly experienced respondents were necessarily ‘more critical’ or more aware of deficiencies is therefore not supported by the data.

After multiplicity adjustment, the revised analyses did not identify robust domain-level differences across professional groups or sport levels. Accordingly, the present data do not provide an empirical basis for deriving separate service models for mass, youth reserve, and elite sport. Although these levels have different clinical and organizational needs, a tiered model should be presented only as a hypothesis for future validation rather than as a direct output of this survey.

Digitalization, workload monitoring, standardized protocols, and intersectoral collaboration remain relevant components of contemporary sports medicine even when they do not occupy the highest positions in a particular stakeholder ranking. The international literature supports the use of structured monitoring and standardized return-to-sport processes while also emphasizing implementation barriers, data quality, privacy, and clinical interpretability [12,13,14,15,16,23]. Their role in Kazakhstan should therefore be assessed through implementation and service-evaluation studies rather than inferred solely from perceived priority scores.

From a management perspective, the findings can be used as preliminary stakeholder input for planning and hypothesis generation. The primary exact-five ranking (*n* = 47) suggests that rehabilitation/recovery, injury prevention, accessibility, financing, workload monitoring, and staffing warrant particular attention in subsequent needs assessments. However, resource allocation decisions require additional evidence on actual service capacity, utilization, regional distribution, workforce supply, costs, and patient or athlete outcomes. The survey should therefore complement, not replace, objective health-system assessments.

The open-ended questions were completed by all respondents, but the original coder-level audit trail and the denominator used for the previously reported thematic frequencies could not be reconstructed with sufficient certainty. Consequently, qualitative percentages and quotations should not be used to strengthen or triangulate the quantitative conclusions until the responses are reanalyzed using a documented coding protocol with clearly specified coders, coder independence, adjudication procedures, definitions of mentions, and denominators.

Several limitations materially constrain generalizability. Recruitment was purposive and supplemented by snowball forwarding, creating a non-probability sample and potential selection bias. The individual recruitment route was not recorded, the total number of people exposed to forwarded survey links was unknown, and nonresponse across the full recruitment network could not be quantified. Nonresponse bias cannot be excluded because the characteristics of nonrespondents were unavailable. The findings therefore cannot be interpreted as representative estimates for all sports-medicine stakeholders in Kazakhstan.

Representation across professional groups was unequal, some respondents reported overlapping professional roles, and several analytical subgroups were small. Regional affiliation was not collected, preventing assessment of geographic coverage or regional heterogeneity. These limitations are particularly important in light of previous national documentation of regional differences in sports-medicine infrastructure and availability [10,11].

Questionnaire administration introduced additional limitations. The Google Forms configuration required a response to the Top-5 item but did not technically restrict respondents to exactly five choices. In addition, the archived study documentation did not permit retrospective verification of whether platform-level controls preventing repeat participation were enabled. Although no duplicate timestamps or exact duplicate response profiles were detected, identity-based duplicate verification was not possible because personal identifiers were not collected. Restricting the primary Top-5 interpretation to the 47 instruction-compliant exact-five responses improves adherence to the intended task definition, but the loss of 53 responses from the primary ranking reduces precision and leaves the resulting hierarchy less robust than it would have been with full procedural compliance. Future surveys should enforce the intended number of selections, document duplicate-prevention settings prospectively, and define a sample-size rationale aligned with the primary analysis.

Finally, the questionnaire was developed specifically for this study and has not undergone full psychometric validation. No evidence of factorial structure, construct validity, test-retest reliability, or discriminatory performance was available. The pronounced ceiling effect and invariant all-maximum response pattern further constrain the ability of the five-point rating block to distinguish between highly prioritized domains and complicate interpretation of high internal-consistency coefficients and subgroup contrasts. These measurement properties substantially restrict the strength of conclusions that can be drawn from the priority ratings, particularly regarding fine distinctions or ordering among domains. Accordingly, the rating results should be treated as preliminary domain-specific stakeholder responses rather than measurements from a validated latent scale. In addition, the inability to reproduce the original qualitative coding process prevents robust mixed-methods interpretation. Future research should refine and formally validate the questionnaire in larger samples with documented regional representation, objective system indicators, prospectively specified subgroup analyses, and independently reproducible qualitative methods.

Overall, the study provides preliminary stakeholder-informed evidence about perceived priorities for future sports medicine development in Kazakhstan. Its main value lies in identifying domains for further representative needs assessment and implementation research, not in demonstrating existing service shortages or establishing a national service framework.

## 5. Conclusions

Among this surveyed non-probability stakeholder sample, the 47 respondents who completed the Top-5 task as instructed most frequently selected rehabilitation/recovery, injury prevention, accessibility of medical support, financing/sustainability, monitoring of training load/recovery, and staffing as development priorities. The all-questionnaire sensitivity ranking (*N* = 100) showed strong but incomplete agreement with the primary exact-five ranking, indicating partial consistency rather than complete stability. The 12-domain ratings exhibited substantial ceiling and invariant all-maximum response patterns that limit discrimination among highly prioritized domains, and the study-specific questionnaire has not undergone full psychometric validation. Accordingly, neither the exact ordering of Top-5 domains nor small differences among five-point ratings should be regarded as robust estimates of a stable priority hierarchy. The findings should therefore be interpreted as perceived priorities within the surveyed sample and not as nationally representative estimates, evidence of current service shortages, or direct national policy priorities. Differences according to professional experience and other subgroup analyses are exploratory and may be influenced by subgroup composition and limited precision. The study does not validate a single composite 12-domain scale, or a tiered national sports-medicine framework. Instead, the results provide preliminary hypotheses for larger studies with documented regional coverage that use more representative sampling, formally validated measurement approaches, and objective measures of service organization, workforce, accessibility, financing, infrastructure, and outcomes.

## Figures and Tables

**Table 1:** Respondent characteristics, analytical role recoding, and questionnaire completion profile (*N* = 100).

Variable	Category	*n*	%
Analytical professional group	Physician	31	31.0
	Physiotherapy/rehabilitation	25	25.0
	Athlete	14	14.0
	Coach	10	10.0
	Manager/administrator	7	7.0
	Multiple professional groups	12	12.0
	Psychologist/other	1	1.0
Level of sport	Mass sport	33	33.0
	Youth reserve sport	27	27.0
	Elite sport	40	40.0
Professional experience	<1 year	4	4.0
	1–3 years	28	28.0
	4–7 years	28	28.0
	8–15 years	22	22.0
	>15 years	18	18.0
Multiple original professional roles	Yes	19	19.0
	No	81	81.0
Top-5 instruction	Exactly five selected	47	47.0
	Not exactly five selected	53	53.0

**Table 2:** Domain-specific priority ratings and proportion of maximum ratings.

Domain	*n*	Median [Q1–Q3]	*n* at 5	% at 5
Accessibility of medical support	100	4.00 [4.00–5.00]	46	46.0
Safety clearance systems	100	5.00 [4.00–5.00]	61	61.0
Digitalization and database management	100	5.00 [3.00–5.00]	56	56.0
Infrastructure and equipment	100	5.00 [4.00–5.00]	52	52.0
Financing and sustainability	100	5.00 [3.00–5.00]	52	52.0
Injury prevention	100	5.00 [4.00–5.00]	51	51.0
Intersectoral collaboration	100	5.00 [3.00–5.00]	57	57.0
Monitoring of training load and recovery	100	4.00 [4.00–5.00]	49	49.0
Standardization of examination protocols	100	5.00 [4.00–5.00]	53	53.0
Rehabilitation and recovery programs	100	5.00 [4.00–5.00]	55	55.0
Return-to-play protocols	100	4.50 [3.75–5.00]	50	50.0
Staffing	100	5.00 [4.00–5.00]	57	57.0

**Table 3:** Primary Top-5 ranking among respondents who selected exactly five domains (*n* = 47).

Rank	Domain	*n*	%	95% Wilson CI
1	Rehabilitation and recovery programs	30	63.8	49.5–76.0%
2	Injury prevention	26	55.3	41.2–68.6%
3	Accessibility of medical support	24	51.1	37.2–64.7%
3	Financing and sustainability	24	51.1	37.2–64.7%
5	Monitoring of training load and recovery	23	48.9	35.3–62.8%
6	Staffing	22	46.8	33.3–60.8%
7	Safety clearance systems	17	36.2	24.0–50.5%
8	Infrastructure and equipment	16	34.0	22.2–48.3%
8	Standardization of examination protocols	16	34.0	22.2–48.3%
10	Digitalization and database management	15	31.9	20.4–46.2%
10	Return-to-play protocols	15	31.9	20.4–46.2%
12	Intersectoral collaboration	7	14.9	7.4–27.7%

**Table 4:** Kruskal–Wallis comparisons of the 12 priority domains across professional-experience groups.

Domain	*H*	*p*	BH-Adjusted *p*	*ε* ^2^	95% Bootstrap CI	Effect
Accessibility of medical support	18.229	0.001	0.005	0.150	0.051–0.331	Moderate
Safety clearance systems	10.991	0.027	0.031	0.074	0.007–0.253	Small
Standardization of examination protocols	10.832	0.029	0.031	0.072	0.007–0.245	Small
Return-to-play protocols	12.745	0.013	0.019	0.092	0.020–0.288	Moderate
Rehabilitation and recovery programs	13.437	0.009	0.016	0.099	0.018–0.293	Moderate
Injury prevention	15.916	0.003	0.009	0.125	0.017–0.338	Moderate
Monitoring of training load and recovery	7.270	0.122	0.122	0.034	—	Small
Staffing	18.029	0.001	0.005	0.148	0.039–0.347	Moderate
Infrastructure and equipment	15.506	0.004	0.009	0.121	0.025–0.311	Moderate
Digitalization and database management	14.760	0.005	0.010	0.113	0.024–0.308	Moderate
Intersectoral collaboration	12.038	0.017	0.023	0.085	0.005–0.271	Moderate
Financing and sustainability	21.648	<0.001	0.003	0.186	0.069–0.382	Moderate

## Data Availability

The raw data are not publicly available due to ethical and confidentiality considerations. Prior authorization from the Ministry of Healthcare of the Republic of Kazakhstan, which funded the study, is required for data sharing. Data may be available from the corresponding author upon reasonable request and subject to the required authorization.

## Supplementary Materials

The following supporting information can be downloaded at: https://www.mdpi.com/article/10.3390/healthcare14193264/s1. Table S1: Full questionnaire and exact response anchors; Table S2: Data quality, completion, and achieved precision; Table S3: Distribution of the number of Top-5 selections; Table S4: Professional-role recoding and exact subgroup sizes; Table S5: Primary Top-5 analysis: exactly five selections; Table S6: All-questionnaire Top-5 sensitivity analysis, rank shifts, and rank agreement; Table S7: Complete Kruskal–Wallis domain-level subgroup analyses; Table S8: Complete Dunn–Holm pairwise comparisons after raw-significant Kruskal–Wallis tests; Table S9: Domain-specific medians by professional-experience group; Table S10: Composition of professional-experience groups and assessment of potential confounding; Table S11: Exploratory Top-5 subgroup tests in the valid exact-five sample; Table S12: Exploratory internal-consistency information; Table S13: Open-ended response completeness and qualitative-analysis status.
