# Supplementary material for: Stakeholder Priorities for Strengthening Sports Medicine Services in Kazakhstan: A Cross-Sectional Health-System Survey
Source: Healthcare (Basel). 2026 Oct 2;14(19):3264. doi: 10.3390/healthcare14193264 (PMC13665269; doi:10.3390/healthcare14193264)
Supplement: Supplementary file 1 [file healthcare-14-03264-s001.zip › healthcare-4492712-supplementary.pdf]

Supplementary Materials

Stakeholder Priorities for Strengthening Sports Medicine Services in Kazakhstan: A Cross-Sectional Health-System Survey

Supplementary Table S1. Full questionnaire and exact response anchors

The questionnaire was administered in Russian through Google Forms. The English wording is supplied for editorial and reviewer use. For items 4–15, the exact instruction was: 'Please rate each area on a 5-point scale, where 1 indicates the lowest priority and 5 indicates the highest priority.' Russian: 'Оцените каждое направление по 5-балльной шкале, где 1 – минимальный приоритет, 5 – максимальный приоритет.'

No.	English translation	Original Russian wording	Response format
1	Respondent specialization	Ваша специализация (обязательно)	Multiple selection: sports physician; general practitioner/internist; ultrasound diagnostics physician; physiotherapist; rehabilitation specialist; coach; athlete; manager/administrator; other
2	Level of sport with which you work	Уровень спорта, с которым вы работаете (обязательно)	Mass sport; youth reserve sport (children/juniors); elite sport; other
3	Experience of work or participation in sport	Стаж работы/занятий в спорте (обязательно)	<1 year; 1–3; 4–7; 8–15; >15 years
4	Accessibility of medical examinations and support, including in regions	Доступность обследований и медицинского сопровождения, в том числе в регионах	1–5 priority rating
5	Safety clearance for training/competition (periodic medical examination, screening)	Безопасность допуска к тренировкам/соревнованиям (УМО, скрининг)	1–5 priority rating
6	Standardization of examination protocols (unified rules, frequency, and scope)	Стандартизация протоколов обследования (единые правила/частота/объём)	1–5 priority rating
7	Availability and implementation of Return-to-Play protocols (return after injury/illness)	Наличие и внедрение протоколов Return-to-Play (возврат после травмы/болезни)	1–5 priority rating
8	Development of rehabilitation and recovery programs	Развитие реабилитации и восстановительных программ	1–5 priority rating
9	Injury prevention (prevention programs and work with risk factors)	Профилактика травм (профилактические программы, работа с факторами риска)	1–5 priority rating
10	Monitoring of load and recovery (control of fatigue/overload)	Мониторинг нагрузки и восстановления (контроль утомления/перегрузки)	1–5 priority rating
11	Staffing capacity (training and staffing: physicians, physiotherapists, nutritionists, etc.)	Кадровое обеспечение (подготовка/штаты: врач, физиотерапевт, нутрициолог и т.д.)	1–5 priority rating
12	Equipment and infrastructure (equipment, laboratory facilities, offices/rooms)	Оснащение и инфраструктура (оборудование, лаборатория, кабинеты)	1–5 priority rating
13	Digitalization and database management (electronic athlete card, injury/load registry)	Цифровизация и база данных (электронная карта спортсмена, регистр травм/нагрузки)	1–5 priority rating
14	Intersectoral collaboration (sport, healthcare, federations)	Межведомственное взаимодействие (спорт – здравоохранение – федерации)	1–5 priority rating
15	Financing and sustainability of the model (sources, tariffs, resource provision)	Финансирование и устойчивость модели (источники, тарифы, ресурсное обеспечение)	1–5 priority rating
16	Select the 5 most important areas (check five options)	Выберите 5 наиболее важных направлений (отметьте пять вариантов)	12-option multiple-choice task; exactly five requested
17	What are the 3 main problems in the current system of medical support for sport?	Какие 3 главные проблемы в текущей системе медицинского сопровождения спорта вы видите?	Open text
18	Which 3 measures should be implemented first (the fastest and most useful)?	Какие 3 меры следует внедрить в первую очередь (самые быстрые и полезные)?	Open text

Top-5 options (exact task): accessibility of examinations; safety clearance; standardization of protocols; Return-to-Play protocols; rehabilitation/recovery; injury prevention; monitoring of load/recovery; staffing; equipment; digitalization/database/registry; intersectoral

collaboration; financing. The Google Forms item was required but was not configured to enforce exactly five selections. The archived study documentation does not allow verification of whether the Google Forms 'Limit to 1 response' setting or mandatory Google-account sign-in was enabled; therefore, technical prevention of repeat participation cannot be confirmed.

Supplementary Table S2. Data quality, completion, and achieved precision

Indicator	Value	Interpretation/status
Number of respondents	100	Total analytical sample
Missing/invalid values across 12 Likert domains	0	Should be 0 for complete Likert block
Correct Top-5: exactly five selected	47	Primary Top-5 analytical sample
Incorrect Top-5: not exactly five selected	53	Retained only for Top-5 sensitivity analysis
Respondents with multiple raw professional roles	19	Role overlap explicitly retained and reported
Respondents spanning multiple analytical professional groups	12	Excluded from profession-based inferential comparisons
Duplicate timestamps	0	Google Forms timestamp check
Possible exact duplicate responses	0	Full-response duplication check from validated dataset
Respondents rating all 12 domains as 5	25	Ceiling/straight-lining indicator
Share of all Likert item responses equal to 5	53.25%	Ceiling-effect indicator
Nominal binomial 95% CI half-width at p = 0.50 (full sample)	±9.8 percentage points	Descriptive achieved precision only; not a design-based margin of error and not an a priori power calculation
Nominal binomial 95% CI half-width at p = 0.50 (valid Top-5 sample)	±14.3 percentage points	Descriptive achieved precision only; not a design-based margin of error and not an a priori power calculation
Incomplete/partially submitted questionnaires	0	Only completed submissions were present; none were excluded for incompleteness before final N=100
Platform-level duplicate-prevention setting	Not verifiable	Archived documentation does not confirm whether 'Limit to 1 response' or mandatory Google-account sign-in was enabled

Supplementary Table S3. Distribution of the number of Top-5 selections

Number selected	n	% of N=100	Analytic status
1	5	5.0%	Not valid for primary Top-5
2	6	6.0%	Not valid for primary Top-5
3	14	14.0%	Not valid for primary Top-5
4	9	9.0%	Not valid for primary Top-5
5	47	47.0%	Valid primary Top-5
6	10	10.0%	Not valid for primary Top-5
7	2	2.0%	Not valid for primary Top-5
8	2	2.0%	Not valid for primary Top-5
9	1	1.0%	Not valid for primary Top-5

Number selected	n	% of N=100	Analytic status
10	1	1.0%	Not valid for primary Top-5
11	1	1.0%	Not valid for primary Top-5
12	2	2.0%	Not valid for primary Top-5

Supplementary Table S4. Professional-role recoding and exact subgroup sizes

If multiple raw roles mapped to the same analytical group, the respondent remained in that group. If roles crossed analytical groups, the respondent was classified as 'multiple professional groups' and excluded only from profession-based inferential testing. The psychologist/other group (n=1) was also excluded from profession-based inference.

Factor	Group	n
Professional experience	<1 year	4
Professional experience	1–3 years	28
Professional experience	4–7 years	28
Professional experience	8–15 years	22
Professional experience	>15 years	18
Level of sport	Mass sport	33
Level of sport	Youth reserve sport	27
Level of sport	Elite sport	40
Professional analytical group - all	Physician	31
Professional analytical group - all	Manager/administrator	7
Professional analytical group - all	Multiple professional groups	12
Professional analytical group - all	Psychologist/other	1
Professional analytical group - all	Athlete	14
Professional analytical group - all	Coach	10
Professional analytical group - all	Physiotherapy/rehabilitation	25
Professional analytical group - inferential sample	Physician	31
Professional analytical group - inferential sample	Manager/administrator	7
Professional analytical group - inferential sample	Athlete	14
Professional analytical group - inferential sample	Coach	10
Professional analytical group - inferential sample	Physiotherapy/rehabilitation	25

Supplementary Table S5. Primary Top-5 analysis: exactly five selections

Rank	Domain	n	N	%	95% Wilson CI
1	Rehabilitation and recovery programs	30	47	63.8%	49.5%–76.0%
2	Injury prevention	26	47	55.3%	41.2%–68.6%

Rank	Domain	n	N	%	95% Wilson CI
3	Accessibility of medical support	24	47	51.1%	37.2%–64.7%
3	Financing and sustainability	24	47	51.1%	37.2%–64.7%
5	Monitoring of training load and recovery	23	47	48.9%	35.3%–62.8%
6	Staffing	22	47	46.8%	33.3%–60.8%
7	Safety clearance systems	17	47	36.2%	24.0%–50.5%
8	Infrastructure and equipment	16	47	34.0%	22.2%–48.3%
8	Standardization of examination protocols	16	47	34.0%	22.2%–48.3%
10	Digitalization and database management	15	47	31.9%	20.4%–46.2%
10	Return-to-play protocols	15	47	31.9%	20.4%–46.2%
12	Intersectoral collaboration	7	47	14.9%	7.4%–27.7%

Supplementary Table S6. All-questionnaire Top-5 sensitivity analysis, rank shifts, and rank agreement

The all-questionnaire analysis is a sensitivity analysis only because 53 respondents did not select exactly five domains.

Domain	Primary rank	Primary %	All-sample rank	All-sample %	Δ percentage points	Rank shift
Rehabilitation and recovery programs	1	63.8%	1	60.0%	-3.8	0
Injury prevention	2	55.3%	1	60.0%	+4.7	-1
Accessibility of medical support	3	51.1%	3	48.0%	-3.1	0
Financing and sustainability	3	51.1%	5	40.0%	-11.1	2
Monitoring of training load and recovery	5	48.9%	8	35.0%	-13.9	3
Staffing	6	46.8%	5	40.0%	-6.8	-1
Safety clearance systems	7	36.2%	4	47.0%	+10.8	-3
Infrastructure and equipment	8	34.0%	10	28.0%	-6.0	2
Standardization of examination protocols	8	34.0%	7	37.0%	+3.0	-1
Digitalization and database management	10	31.9%	10	28.0%	-3.9	0
Return-to-play protocols	10	31.9%	9	34.0%	+2.1	-1
Intersectoral collaboration	12	14.9%	12	17.0%	+2.1	0

Agreement statistic	Estimate	p	Interpretation
Spearman rank correlation	0.889	<0.001	Strong rank agreement does not imply identical rankings
Kendall rank correlation (ties accounted for)	0.762	<0.001	Agreement should be described as strong/partial rather than complete stability

Supplementary Table S7. Complete Kruskal–Wallis domain-level subgroup analyses

BH-adjusted p-values control multiplicity across the 12 domain tests within each grouping factor. Bootstrap 95% confidence intervals for ε^2 were calculated for raw-significant omnibus tests using 2,000 stratified bootstrap resamples. Subgroup analyses are exploratory.

Factor	Domain	n	k	H	p	BH p	BH sig.	ε²	95% CI	Effect
Professional group	Accessibility of medical support	87	5	5.290	0.259	0.777	No	0.016		Small
Professional group	Safety clearance systems	87	5	6.472	0.167	0.777	No	0.030		Small
Professional group	Standardization of examination protocols	87	5	6.599	0.159	0.777	No	0.032		Small
Professional group	Return-to-play protocols	87	5	2.822	0.588	0.918	No	0.000		Negligible
Professional group	Rehabilitation and recovery programs	87	5	2.580	0.630	0.918	No	0.000		Negligible
Professional group	Injury prevention	87	5	1.840	0.765	0.918	No	0.000		Negligible
Professional group	Monitoring of training load and recovery	87	5	2.008	0.734	0.918	No	0.000		Negligible
Professional group	Staffing	87	5	1.045	0.903	0.976	No	0.000		Negligible
Professional group	Infrastructure and equipment	87	5	5.374	0.251	0.777	No	0.017		Small
Professional group	Digitalization and database management	87	5	3.586	0.465	0.918	No	0.000		Negligible
Professional group	Intersectoral collaboration	87	5	2.131	0.712	0.918	No	0.000		Negligible
Professional group	Financing and sustainability	87	5	0.476	0.976	0.976	No	0.000		Negligible
Level of sport	Accessibility of medical support	100	3	0.488	0.783	0.855	No	0.000		Negligible
Level of sport	Safety clearance systems	100	3	2.140	0.343	0.457	No	0.001		Negligible
Level of sport	Standardization of examination protocols	100	3	1.073	0.585	0.702	No	0.000		Negligible
Level of sport	Return-to-play protocols	100	3	0.171	0.918	0.918	No	0.000		Negligible
Level of sport	Rehabilitation and recovery programs	100	3	4.218	0.121	0.324	No	0.023		Small
Level of sport	Injury prevention	100	3	5.662	0.059	0.324	No	0.038		Small
Level of sport	Monitoring of training load and recovery	100	3	3.606	0.165	0.330	No	0.017		Small
Level of sport	Staffing	100	3	2.265	0.322	0.457	No	0.003		Negligible
Level of sport	Infrastructure and equipment	100	3	7.708	0.021	0.254	No	0.059	0.001–0.183	Small
Level of sport	Digitalization and database management	100	3	4.738	0.094	0.324	No	0.028		Small
Level of sport	Intersectoral collaboration	100	3	4.005	0.135	0.324	No	0.021		Small
Level of sport	Financing and sustainability	100	3	2.946	0.229	0.393	No	0.010		Negligible
Professional experience	Accessibility of medical support	100	5	18.229	0.001	0.005	Yes	0.150	0.051–0.331	Moderate
Professional experience	Safety clearance systems	100	5	10.991	0.027	0.031	Yes	0.074	0.007–0.253	Small
Professional experience	Standardization of examination protocols	100	5	10.832	0.029	0.031	Yes	0.072	0.007–0.245	Small
Professional experience	Return-to-play protocols	100	5	12.745	0.013	0.019	Yes	0.092	0.020–0.288	Moderate
Professional experience	Rehabilitation and recovery programs	100	5	13.437	0.009	0.016	Yes	0.099	0.018–0.293	Moderate

Factor	Domain	n	k	H	p	BH p	BH sig.	ε²	95% CI	Effect
Professional experience	Injury prevention	100	5	15.916	0.003	0.009	Yes	0.125	0.017–0.338	Moderate
Professional experience	Monitoring of training load and recovery	100	5	7.270	0.122	0.122	No	0.034		Small
Professional experience	Staffing	100	5	18.029	0.001	0.005	Yes	0.148	0.039–0.347	Moderate
Professional experience	Infrastructure and equipment	100	5	15.506	0.004	0.009	Yes	0.121	0.025–0.311	Moderate
Professional experience	Digitalization and database management	100	5	14.760	0.005	0.010	Yes	0.113	0.024–0.308	Moderate
Professional experience	Intersectoral collaboration	100	5	12.038	0.017	0.023	Yes	0.085	0.005–0.271	Moderate
Professional experience	Financing and sustainability	100	5	21.648	<0.001	0.003	Yes	0.186	0.069–0.382	Moderate

Supplementary Table S8. Complete Dunn–Holm pairwise comparisons after raw-significant Kruskal–Wallis tests

All adjusted pairwise p-values are shown. For level of sport, the infrastructure/equipment omnibus test was raw-significant but did not remain significant after BH correction (BH p=0.254); its pairwise results are therefore descriptive and are not interpreted as multiplicity-controlled evidence in the manuscript.

Factor	Domain	Group 1	Group 2	z	Raw p	Holm p	Pair sig.	Omnibus BH p	Omnibus ε²
Level of sport	Infrastructure and equipment	Mass sport	Youth reserve sport	2.567	0.010	0.031	Yes	0.254	0.059
Level of sport	Infrastructure and equipment	Mass sport	Elite sport	0.356	0.722	0.722	No	0.254	0.059
Level of sport	Infrastructure and equipment	Youth reserve sport	Elite sport	-2.338	0.019	0.039	Yes	0.254	0.059
Professional experience	Accessibility of medical support	<1 year	1–3 years	-1.569	0.117	0.505	No	0.005	0.150
Professional experience	Accessibility of medical support	<1 year	4–7 years	-2.389	0.017	0.101	No	0.005	0.150
Professional experience	Accessibility of medical support	<1 year	8–15 years	-0.986	0.324	0.828	No	0.005	0.150
Professional experience	Accessibility of medical support	<1 year	>15 years	-2.906	0.004	0.033	Yes	0.005	0.150
Professional experience	Accessibility of medical support	1–3 years	4–7 years	-1.640	0.101	0.505	No	0.005	0.150
Professional experience	Accessibility of medical support	1–3 years	8–15 years	1.063	0.288	0.828	No	0.005	0.150
Professional experience	Accessibility of medical support	1–3 years	>15 years	-2.540	0.011	0.078	No	0.005	0.150
Professional experience	Accessibility of medical support	4–7 years	8–15 years	2.602	0.009	0.074	No	0.005	0.150
Professional experience	Accessibility of medical support	4–7 years	>15 years	-1.089	0.276	0.828	No	0.005	0.150
Professional experience	Accessibility of medical support	8–15 years	>15 years	-3.367	<0.001	0.008	Yes	0.005	0.150
Professional experience	Safety clearance systems	<1 year	1–3 years	-1.203	0.229	0.916	No	0.031	0.074
Professional experience	Safety clearance systems	<1 year	4–7 years	-1.878	0.060	0.363	No	0.031	0.074
Professional experience	Safety clearance systems	<1 year	8–15 years	-0.762	0.446	1.000	No	0.031	0.074
Professional experience	Safety clearance systems	<1 year	>15 years	-2.230	0.026	0.232	No	0.031	0.074
Professional experience	Safety clearance systems	1–3 years	4–7 years	-1.350	0.177	0.885	No	0.031	0.074
Professional experience	Safety clearance systems	1–3 years	8–15 years	0.803	0.422	1.000	No	0.031	0.074
Professional experience	Safety clearance systems	1–3 years	>15 years	-1.952	0.051	0.356	No	0.031	0.074
Professional experience	Safety clearance systems	4–7 years	8–15 years	2.069	0.039	0.308	No	0.031	0.074
Professional experience	Safety clearance systems	4–7 years	>15 years	-0.758	0.448	1.000	No	0.031	0.074

Factor	Domain	Group 1	Group 2	z	Raw p	Holm p	Pair sig.	Omnibus BH p	Omnibus ε²
Professional experience	Safety clearance systems	8–15 years	>15 years	-2.576	0.010	0.100	No	0.031	0.074
Professional experience	Standardization of examination protocols	<1 year	1–3 years	-1.488	0.137	0.684	No	0.031	0.072
Professional experience	Standardization of examination protocols	<1 year	4–7 years	-1.665	0.096	0.576	No	0.031	0.072
Professional experience	Standardization of examination protocols	<1 year	8–15 years	-1.246	0.213	0.852	No	0.031	0.072
Professional experience	Standardization of examination protocols	<1 year	>15 years	-2.690	0.007	0.071	No	0.031	0.072
Professional experience	Standardization of examination protocols	1–3 years	4–7 years	-0.354	0.724	1.000	No	0.031	0.072
Professional experience	Standardization of examination protocols	1–3 years	8–15 years	0.415	0.678	1.000	No	0.031	0.072
Professional experience	Standardization of examination protocols	1–3 years	>15 years	-2.290	0.022	0.176	No	0.031	0.072
Professional experience	Standardization of examination protocols	4–7 years	8–15 years	0.747	0.455	1.000	No	0.031	0.072
Professional experience	Standardization of examination protocols	4–7 years	>15 years	-1.977	0.048	0.336	No	0.031	0.072
Professional experience	Standardization of examination protocols	8–15 years	>15 years	-2.548	0.011	0.097	No	0.031	0.072
Professional experience	Return-to-play protocols	<1 year	1–3 years	-0.217	0.828	1.000	No	0.019	0.092
Professional experience	Return-to-play protocols	<1 year	4–7 years	-0.632	0.527	1.000	No	0.019	0.092
Professional experience	Return-to-play protocols	<1 year	8–15 years	0.251	0.802	1.000	No	0.019	0.092
Professional experience	Return-to-play protocols	<1 year	>15 years	-1.697	0.090	0.628	No	0.019	0.092
Professional experience	Return-to-play protocols	1–3 years	4–7 years	-0.831	0.406	1.000	No	0.019	0.092
Professional experience	Return-to-play protocols	1–3 years	8–15 years	0.885	0.376	1.000	No	0.019	0.092
Professional experience	Return-to-play protocols	1–3 years	>15 years	-2.721	0.007	0.059	No	0.019	0.092
Professional experience	Return-to-play protocols	4–7 years	8–15 years	1.664	0.096	0.628	No	0.019	0.092
Professional experience	Return-to-play protocols	4–7 years	>15 years	-1.986	0.047	0.376	No	0.019	0.092
Professional experience	Return-to-play protocols	8–15 years	>15 years	-3.380	<0.001	0.007	Yes	0.019	0.092
Professional experience	Rehabilitation and recovery programs	<1 year	1–3 years	-0.229	0.819	1.000	No	0.016	0.099
Professional experience	Rehabilitation and recovery programs	<1 year	4–7 years	-0.004	0.997	1.000	No	0.016	0.099
Professional experience	Rehabilitation and recovery programs	<1 year	8–15 years	-0.043	0.966	1.000	No	0.016	0.099
Professional experience	Rehabilitation and recovery programs	<1 year	>15 years	-1.799	0.072	0.504	No	0.016	0.099
Professional experience	Rehabilitation and recovery programs	1–3 years	4–7 years	0.450	0.652	1.000	No	0.016	0.099
Professional experience	Rehabilitation and recovery programs	1–3 years	8–15 years	0.348	0.728	1.000	No	0.016	0.099
Professional experience	Rehabilitation and recovery programs	1–3 years	>15 years	-2.887	0.004	0.031	Yes	0.016	0.099
Professional experience	Rehabilitation and recovery programs	4–7 years	8–15 years	-0.075	0.940	1.000	No	0.016	0.099
Professional experience	Rehabilitation and recovery programs	4–7 years	>15 years	-3.285	0.001	0.010	Yes	0.016	0.099
Professional experience	Rehabilitation and recovery programs	8–15 years	>15 years	-3.055	0.002	0.020	Yes	0.016	0.099
Professional experience	Injury prevention	<1 year	1–3 years	-0.318	0.751	1.000	No	0.009	0.125
Professional experience	Injury prevention	<1 year	4–7 years	-0.241	0.809	1.000	No	0.009	0.125

Factor	Domain	Group 1	Group 2	z	Raw p	Holm p	Pair sig.	Omnibus BH p	Omnibus ε^2
Professional experience	Injury prevention	<1 year	8–15 years	-0.052	0.958	1.000	No	0.009	0.125
Professional experience	Injury prevention	<1 year	>15 years	-2.059	0.040	0.277	No	0.009	0.125
Professional experience	Injury prevention	1–3 years	4–7 years	0.153	0.879	1.000	No	0.009	0.125
Professional experience	Injury prevention	1–3 years	8–15 years	0.496	0.620	1.000	No	0.009	0.125
Professional experience	Injury prevention	1–3 years	>15 years	-3.205	0.001	0.011	Yes	0.009	0.125
Professional experience	Injury prevention	4–7 years	8–15 years	0.353	0.724	1.000	No	0.009	0.125
Professional experience	Injury prevention	4–7 years	>15 years	-3.340	<0.001	0.008	Yes	0.009	0.125
Professional experience	Injury prevention	8–15 years	>15 years	-3.491	<0.001	0.005	Yes	0.009	0.125
Professional experience	Staffing	<1 year	1–3 years	-0.997	0.319	1.000	No	0.005	0.148
Professional experience	Staffing	<1 year	4–7 years	-1.633	0.102	0.614	No	0.005	0.148
Professional experience	Staffing	<1 year	8–15 years	-1.145	0.252	1.000	No	0.005	0.148
Professional experience	Staffing	<1 year	>15 years	-2.966	0.003	0.024	Yes	0.005	0.148
Professional experience	Staffing	1–3 years	4–7 years	-1.273	0.203	1.000	No	0.005	0.148
Professional experience	Staffing	1–3 years	8–15 years	-0.314	0.753	1.000	No	0.005	0.148
Professional experience	Staffing	1–3 years	>15 years	-3.664	<0.001	0.002	Yes	0.005	0.148
Professional experience	Staffing	4–7 years	8–15 years	0.880	0.379	1.000	No	0.005	0.148
Professional experience	Staffing	4–7 years	>15 years	-2.538	0.011	0.078	No	0.005	0.148
Professional experience	Staffing	8–15 years	>15 years	-3.201	0.001	0.012	Yes	0.005	0.148
Professional experience	Infrastructure and equipment	<1 year	1–3 years	0.355	0.723	1.000	No	0.009	0.121
Professional experience	Infrastructure and equipment	<1 year	4–7 years	-0.412	0.681	1.000	No	0.009	0.121
Professional experience	Infrastructure and equipment	<1 year	8–15 years	-0.052	0.959	1.000	No	0.009	0.121
Professional experience	Infrastructure and equipment	<1 year	>15 years	-1.740	0.082	0.573	No	0.009	0.121
Professional experience	Infrastructure and equipment	1–3 years	4–7 years	-1.533	0.125	0.751	No	0.009	0.121
Professional experience	Infrastructure and equipment	1–3 years	8–15 years	-0.765	0.444	1.000	No	0.009	0.121
Professional experience	Infrastructure and equipment	1–3 years	>15 years	-3.812	<0.001	0.001	Yes	0.009	0.121
Professional experience	Infrastructure and equipment	4–7 years	8–15 years	0.673	0.501	1.000	No	0.009	0.121
Professional experience	Infrastructure and equipment	4–7 years	>15 years	-2.456	0.014	0.113	No	0.009	0.121
Professional experience	Infrastructure and equipment	8–15 years	>15 years	-2.938	0.003	0.030	Yes	0.009	0.121
Professional experience	Digitalization and database management	<1 year	1–3 years	-0.455	0.649	1.000	No	0.010	0.113
Professional experience	Digitalization and database management	<1 year	4–7 years	-0.780	0.435	1.000	No	0.010	0.113
Professional experience	Digitalization and database management	<1 year	8–15 years	-0.665	0.506	1.000	No	0.010	0.113
Professional experience	Digitalization and database management	<1 year	>15 years	-2.338	0.019	0.136	No	0.010	0.113
Professional experience	Digitalization and database management	1–3 years	4–7 years	-0.651	0.515	1.000	No	0.010	0.113

Factor	Domain	Group 1	Group 2	z	Raw p	Holm p	Pair sig.	Omnibus BH p	Omnibus ε²
Professional experience	Digitalization and database management	1–3 years	8–15 years	-0.416	0.678	1.000	No	0.010	0.113
Professional experience	Digitalization and database management	1–3 years	>15 years	-3.473	<0.001	0.005	Yes	0.010	0.113
Professional experience	Digitalization and database management	4–7 years	8–15 years	0.195	0.845	1.000	No	0.010	0.113
Professional experience	Digitalization and database management	4–7 years	>15 years	-2.897	0.004	0.031	Yes	0.010	0.113
Professional experience	Digitalization and database management	8–15 years	>15 years	-2.929	0.003	0.031	Yes	0.010	0.113
Professional experience	Intersectoral collaboration	<1 year	1–3 years	-0.158	0.875	1.000	No	0.023	0.085
Professional experience	Intersectoral collaboration	<1 year	4–7 years	-0.712	0.476	1.000	No	0.023	0.085
Professional experience	Intersectoral collaboration	<1 year	8–15 years	-0.269	0.788	1.000	No	0.023	0.085
Professional experience	Intersectoral collaboration	<1 year	>15 years	-1.886	0.059	0.415	No	0.023	0.085
Professional experience	Intersectoral collaboration	1–3 years	4–7 years	-1.109	0.267	1.000	No	0.023	0.085
Professional experience	Intersectoral collaboration	1–3 years	8–15 years	-0.217	0.828	1.000	No	0.023	0.085
Professional experience	Intersectoral collaboration	1–3 years	>15 years	-3.171	0.002	0.015	Yes	0.023	0.085
Professional experience	Intersectoral collaboration	4–7 years	8–15 years	0.824	0.410	1.000	No	0.023	0.085
Professional experience	Intersectoral collaboration	4–7 years	>15 years	-2.190	0.029	0.228	No	0.023	0.085
Professional experience	Intersectoral collaboration	8–15 years	>15 years	-2.820	0.005	0.043	Yes	0.023	0.085
Professional experience	Financing and sustainability	<1 year	1–3 years	0.249	0.804	1.000	No	0.003	0.186
Professional experience	Financing and sustainability	<1 year	4–7 years	-0.155	0.877	1.000	No	0.003	0.186
Professional experience	Financing and sustainability	<1 year	8–15 years	0.460	0.645	1.000	No	0.003	0.186
Professional experience	Financing and sustainability	<1 year	>15 years	-1.962	0.050	0.348	No	0.003	0.186
Professional experience	Financing and sustainability	1–3 years	4–7 years	-0.807	0.420	1.000	No	0.003	0.186
Professional experience	Financing and sustainability	1–3 years	8–15 years	0.412	0.680	1.000	No	0.003	0.186
Professional experience	Financing and sustainability	1–3 years	>15 years	-4.030	<0.001	<0.001	Yes	0.003	0.186
Professional experience	Financing and sustainability	4–7 years	8–15 years	1.169	0.242	1.000	No	0.003	0.186
Professional experience	Financing and sustainability	4–7 years	>15 years	-3.316	<0.001	0.007	Yes	0.003	0.186
Professional experience	Financing and sustainability	8–15 years	>15 years	-4.200	<0.001	<0.001	Yes	0.003	0.186

Supplementary Table S9. Domain-specific medians by professional-experience group

Domain	Experience group	n	Median	Q1	Q3
Accessibility of medical support	<1 year	4	5.00	5.00	5.00
Safety clearance systems	<1 year	4	5.00	5.00	5.00
Digitalization and database management	<1 year	4	5.00	4.75	5.00
Infrastructure and equipment	<1 year	4	4.50	4.00	5.00
Financing and sustainability	<1 year	4	4.50	3.75	5.00

Domain	Experience group	n	Median	Q1	Q3
Injury prevention	<1 year	4	5.00	4.25	5.00
Intersectoral collaboration	<1 year	4	5.00	4.50	5.00
Monitoring of training load and recovery	<1 year	4	5.00	4.50	5.00
Standardization of examination protocols	<1 year	4	5.00	5.00	5.00
Rehabilitation and recovery programs	<1 year	4	5.00	4.25	5.00
Return-to-play protocols	<1 year	4	5.00	4.25	5.00
Staffing	<1 year	4	5.00	5.00	5.00
Accessibility of medical support	1–3 years	28	4.50	4.00	5.00
Safety clearance systems	1–3 years	28	5.00	4.00	5.00
Digitalization and database management	1–3 years	28	5.00	4.00	5.00
Infrastructure and equipment	1–3 years	28	5.00	4.00	5.00
Financing and sustainability	1–3 years	28	5.00	4.00	5.00
Injury prevention	1–3 years	28	5.00	4.00	5.00
Intersectoral collaboration	1–3 years	28	5.00	4.00	5.00
Monitoring of training load and recovery	1–3 years	28	5.00	4.00	5.00
Standardization of examination protocols	1–3 years	28	5.00	4.00	5.00
Rehabilitation and recovery programs	1–3 years	28	5.00	4.00	5.00
Return-to-play protocols	1–3 years	28	5.00	4.00	5.00
Staffing	1–3 years	28	5.00	4.00	5.00
Accessibility of medical support	4–7 years	28	4.00	3.00	5.00
Safety clearance systems	4–7 years	28	4.00	4.00	5.00
Digitalization and database management	4–7 years	28	5.00	3.75	5.00
Infrastructure and equipment	4–7 years	28	4.50	3.75	5.00
Financing and sustainability	4–7 years	28	5.00	3.00	5.00
Injury prevention	4–7 years	28	5.00	4.00	5.00
Intersectoral collaboration	4–7 years	28	5.00	3.00	5.00
Monitoring of training load and recovery	4–7 years	28	4.00	3.00	5.00
Standardization of examination protocols	4–7 years	28	4.50	4.00	5.00
Rehabilitation and recovery programs	4–7 years	28	5.00	4.00	5.00
Return-to-play protocols	4–7 years	28	4.00	3.75	5.00
Staffing	4–7 years	28	4.00	4.00	5.00

Domain	Experience group	n	Median	Q1	Q3
Accessibility of medical support	8–15 years	22	5.00	4.00	5.00
Safety clearance systems	8–15 years	22	5.00	5.00	5.00
Digitalization and database management	8–15 years	22	5.00	3.25	5.00
Infrastructure and equipment	8–15 years	22	5.00	4.00	5.00
Financing and sustainability	8–15 years	22	5.00	4.25	5.00
Injury prevention	8–15 years	22	5.00	4.00	5.00
Intersectoral collaboration	8–15 years	22	5.00	3.25	5.00
Monitoring of training load and recovery	8–15 years	22	5.00	4.00	5.00
Standardization of examination protocols	8–15 years	22	5.00	4.00	5.00
Rehabilitation and recovery programs	8–15 years	22	5.00	4.00	5.00
Return-to-play protocols	8–15 years	22	5.00	4.25	5.00
Staffing	8–15 years	22	5.00	3.50	5.00
Accessibility of medical support	>15 years	18	3.00	3.00	4.75
Safety clearance systems	>15 years	18	4.00	3.00	5.00
Digitalization and database management	>15 years	18	3.00	2.25	4.00
Infrastructure and equipment	>15 years	18	3.00	2.00	4.00
Financing and sustainability	>15 years	18	2.00	2.00	3.00
Injury prevention	>15 years	18	2.50	2.00	4.00
Intersectoral collaboration	>15 years	18	3.00	2.00	4.75
Monitoring of training load and recovery	>15 years	18	4.00	2.25	4.75
Standardization of examination protocols	>15 years	18	3.50	2.25	5.00
Rehabilitation and recovery programs	>15 years	18	3.50	2.00	4.00
Return-to-play protocols	>15 years	18	3.50	3.00	4.00
Staffing	>15 years	18	2.50	1.25	4.75

Supplementary Table S10. Composition of professional-experience groups and assessment of potential confounding

Association	n	Test	p	Cramer's V	Minimum expected
Experience × level of sport	100	Fisher exact, Monte Carlo B=1e+05	0.0022	0.331	1.08
Experience × professional group	87	Fisher exact, Monte Carlo B=1e+05	0.102	0.250	0.32

Composition of the >15-year group (n=18):

Dimension	Category	n	N	% within >15-year group
Level of sport	Mass sport	4	18	22.2%
Level of sport	Youth reserve sport	2	18	11.1%
Level of sport	Elite sport	12	18	66.7%
Professional analytical group	Physician	3	18	16.7%
Professional analytical group	Manager/administrator	2	18	11.1%
Professional analytical group	Multiple professional groups	2	18	11.1%
Professional analytical group	Athlete	1	18	5.6%
Professional analytical group	Coach	2	18	11.1%
Professional analytical group	Physiotherapy/rehabilitation	8	18	44.4%

Supplementary Table S11. Exploratory Top-5 subgroup tests in the valid exact-five sample

All tests use only respondents who selected exactly five domains. Profession-based testing used n=41 because groups with fewer than five valid Top-5 respondents, multiple-group respondents, and psychologist/other were excluded. No domain was significant after BH correction in any grouping factor.

Factor	Domain	n	Test	p	BH p	BH sig.	Cramer's V	Min expected
Professional group	Accessibility of medical support	41	Fisher exact, Monte Carlo B=1e+05	0.296	0.641	No	0.301	2.32
Professional group	Safety clearance systems	41	Fisher exact, Monte Carlo B=1e+05	0.677	0.796	No	0.217	1.59
Professional group	Standardization of examination protocols	41	Fisher exact, Monte Carlo B=1e+05	0.028	0.237	No	0.457	1.46
Professional group	Return-to-play protocols	41	Fisher exact, Monte Carlo B=1e+05	0.040	0.237	No	0.438	1.46
Professional group	Rehabilitation and recovery programs	41	Fisher exact, Monte Carlo B=1e+05	0.348	0.641	No	0.292	1.71
Professional group	Injury prevention	41	Fisher exact, Monte Carlo B=1e+05	0.796	0.796	No	0.187	2.07
Professional group	Monitoring of training load and recovery	41	Fisher exact, Monte Carlo B=1e+05	0.758	0.796	No	0.174	2.44
Professional group	Staffing	41	Fisher exact, Monte Carlo B=1e+05	0.420	0.641	No	0.273	2.20
Professional group	Infrastructure and equipment	41	Fisher exact, Monte Carlo B=1e+05	0.128	0.512	No	0.376	1.71
Professional group	Digitalization and database management	41	Fisher exact, Monte Carlo B=1e+05	0.427	0.641	No	0.276	1.83
Professional group	Intersectoral collaboration	41	Fisher exact, Monte Carlo B=1e+05	0.298	0.641	No	0.290	0.85
Professional group	Financing and sustainability	41	Fisher exact, Monte Carlo B=1e+05	0.540	0.719	No	0.245	2.44
Level of sport	Accessibility of medical support	47	Pearson chi-square	0.582	0.776	No	0.152	5.38
Level of sport	Safety clearance systems	47	Fisher exact, Monte Carlo B=1e+05	0.742	0.781	No	0.117	3.98
Level of sport	Standardization of examination protocols	47	Fisher exact, Monte Carlo B=1e+05	0.570	0.776	No	0.153	3.74
Level of sport	Return-to-play protocols	47	Fisher exact, Monte Carlo B=1e+05	0.091	0.776	No	0.313	3.51
Level of sport	Rehabilitation and recovery programs	47	Fisher exact, Monte Carlo B=1e+05	0.287	0.776	No	0.224	3.98
Level of sport	Injury prevention	47	Fisher exact, Monte Carlo B=1e+05	0.417	0.776	No	0.215	4.91
Level of sport	Monitoring of training load and recovery	47	Pearson chi-square	0.167	0.776	No	0.276	5.38
Level of sport	Staffing	47	Pearson chi-square	0.577	0.776	No	0.153	5.15

Factor	Domain	n	Test	p	BH p	BH sig.	Cramer's V	Min expected
Level of sport	Infrastructure and equipment	47	Fisher exact, Monte Carlo B=1e+05	0.451	0.776	No	0.189	3.74
Level of sport	Digitalization and database management	47	Fisher exact, Monte Carlo B=1e+05	0.781	0.781	No	0.094	3.51
Level of sport	Intersectoral collaboration	47	Fisher exact, Monte Carlo B=1e+05	0.575	0.776	No	0.156	1.64
Level of sport	Financing and sustainability	47	Pearson chi-square	0.764	0.781	No	0.107	5.38
Professional experience, collapsed	Accessibility of medical support	47	Fisher exact, Monte Carlo B=1e+05	0.856	0.856	No	0.147	4.40
Professional experience, collapsed	Safety clearance systems	47	Fisher exact, Monte Carlo B=1e+05	0.628	0.763	No	0.203	3.26
Professional experience, collapsed	Standardization of examination protocols	47	Fisher exact, Monte Carlo B=1e+05	0.457	0.763	No	0.242	3.06
Professional experience, collapsed	Return-to-play protocols	47	Fisher exact, Monte Carlo B=1e+05	0.047	0.334	No	0.421	2.87
Professional experience, collapsed	Rehabilitation and recovery programs	47	Fisher exact, Monte Carlo B=1e+05	0.652	0.763	No	0.203	3.26
Professional experience, collapsed	Injury prevention	47	Fisher exact, Monte Carlo B=1e+05	0.535	0.763	No	0.230	4.02
Professional experience, collapsed	Monitoring of training load and recovery	47	Fisher exact, Monte Carlo B=1e+05	0.061	0.334	No	0.397	4.40
Professional experience, collapsed	Staffing	47	Fisher exact, Monte Carlo B=1e+05	0.209	0.560	No	0.314	4.21
Professional experience, collapsed	Infrastructure and equipment	47	Fisher exact, Monte Carlo B=1e+05	0.699	0.763	No	0.173	3.06
Professional experience, collapsed	Digitalization and database management	47	Fisher exact, Monte Carlo B=1e+05	0.496	0.763	No	0.233	2.87
Professional experience, collapsed	Intersectoral collaboration	47	Fisher exact, Monte Carlo B=1e+05	0.233	0.560	No	0.302	1.34
Professional experience, collapsed	Financing and sustainability	47	Fisher exact, Monte Carlo B=1e+05	0.084	0.334	No	0.384	4.40

Supplementary Table S12. Exploratory internal-consistency information

Indicator	Value
Cronbach alpha	0.944
Standardized Cronbach alpha	0.945
Average inter-item correlation	0.590
Interpretation	Exploratory internal-consistency evidence only. It does not establish unidimensionality or justify a composite scale score.

Interpretive limitation: Cronbach's alpha describes internal consistency of the response pattern only. It does not establish unidimensionality, factorial validity, or the appropriateness of a composite 12-domain score. No total or mean composite score is used for inferential conclusions in the revised manuscript.

Supplementary Table S13. Open-ended response completeness and qualitative-analysis status

Question	n answered	N total	Completion	Status
Three main problems in the current system	100	100	100.0%	Respondent denominator. Theme frequencies require independent coding and are not generated here.
Three measures to implement first	100	100	100.0%	Respondent denominator. Theme frequencies require independent coding and are not generated here.

The original thematic-frequency table is not reproduced because the archived materials did not permit verification of coder number, coder independence, coding adjudication, or the denominator used for thematic percentages. A separate qualitative re-analysis with a documented coding protocol and independent coders is recommended before any thematic frequencies or representative quotations are reported.
